# Combined Effects of the North Atlantic Oscillation and the Arctic Oscillation on Sea Surface Temperature in the Alborán Sea

**DOI:** 10.1371/journal.pone.0062201

**Published:** 2013-04-18

**Authors:** José C. Báez, Luis Gimeno, Moncho Gómez-Gesteira, Francisco Ferri-Yáñez, Raimundo Real

**Affiliations:** 1 Universidad de Málaga, Departamento Biología Animal, Málaga, Spain; 2 Instituto Español de Oceanografía, Centro Oceanográfico de Málaga, Fuengirola, Málaga, Spain; 3 Universidad de Vigo, Facultad de Ciencias de Ourense, Ourense, Spain; 4 CSIC (Consejo Superior de Investigaciones Científicas), Museo de Ciencias Naturales, Madrid, Spain; University of Vigo, Spain

## Abstract

We explored the possible effects of the North Atlantic Oscillation (NAO) and Arctic Oscillation (AO) on interannual sea surface temperature (SST) variations in the Alborán Sea, both separately and combined. The probability of observing mean annual SST values higher than average was related to NAO and AO values of the previous year. The effect of NAO on SST was negative, while that of AO was positive. The pure effects of NAO and AO on SST are obscuring each other, due to the positive correlation between them. When decomposing SST, NAO and AO in seasonal values, we found that variation in mean annual SST and mean winter SST was significantly related to the mean autumn NAO of the previous year, while mean summer SST was related to mean autumn AO of the previous year. The one year delay in the effect of the NAO and AO on the SST could be partially related to the amount of accumulated snow, as we found a significant correlation between the total snow in the North Alborán watershed for a year with the annual average SST of the subsequent year. A positive AO implies a colder atmosphere in the Polar Regions, which could favour occasional cold waves over the Iberian Peninsula which, when coupled with precipitations favoured by a negative NAO, may result in snow precipitation. This snow may be accumulated in the high peaks and melt down in spring-summer of the following year, which consequently increases the runoff of freshwater to the sea, which in turn causes a diminution of sea surface salinity and density, and blocks the local upwelling of colder water, resulting in a higher SST.

## Introduction

The most important mechanism responsible for interannual climate variability in South-West Europe is the North Atlantic Oscillation (NAO), particularly in winter [1]–[8]. The NAO reflects fluctuations in atmospheric pressure at sea-level between the Icelandic Low and the High of Azores. The NAO is associated with many meteorological variations in the North Atlantic region, affecting wind speed and direction and differences in temperature and rainfall [2]–[3]. Usually, NAO indices are defined as the difference of surface pressure between two stations placed at similar longitude (in the band 5°W–30°W) but different latitude, with one placed at high latitudes (usually in the band 60°N–70°N) and the other at subtropical latitudes (usually in the band 35°N–40°N) [5]–[6].The NAO index can be positive or negative. It is widely known that the positive phases of NAO induce higher than average westerly winds across northern mid-latitudes with a dry climate on the Iberian Peninsula, while the negative phases of NAO induce major precipitation in southern Europe 1.

The NAO is not the only climatic index correlated with interannual climate variability in the Northern Hemisphere. The Arctic Oscillation (AO) is a climate index that may range from positive to negative values according to pressure anomalies in the Arctic region. Thompson and Wallace [9] suggested that the AO is characterized by a meridional dipole in sea level atmospheric pressure between Polar Regions and mid-latitudes, and could be interpreted as the surface signature of modulations in the strength of the polar vortex aloft [9]. When the AO index is positive (characterized by a strengthening of the polar vortex), surface pressure is low in the polar region, and the opposite occurs when the index is negative.

Since the AO was introduced, there has been a vivid debate about its physical reality, and its connection with the NAO [10]. Although recent studies have highlighted the strong relationship between NAO and AO [11]–[15], currently there is no consensus on the links between them [14]. Both the NAO and the AO may be either reflections in the troposphere from the same common cause [9], [16]–[7] or different phenomena with independent and complementary effects [13]. There has been significant differences in the way indices used to represent NAO and AO are calculated. NAO has been usually calculated as difference of surface pressure in two stations one placed in the surrounding of 30°N and the other closed to 60°N. On the other hand, AO indices has been always based on Principal Components (PCs) from an Empirical Orthogonal Function (EOF) analysis of the surface pressure or in the geopotential fields close to the surface (typically 1000 hPA) in a grid domain typically from 20°N to 70°N [18]. Currently the NAO index calculation is widely extended based upon the Rotated Principal Component Analysis (RPCA) used by Barnston and Livezey [6]. The RPCA technique is applied to monthly standardised 500-mb height anomalies in the region 20°N–90°N. This way of calculating the NAO index is quite similar to the way that the AO index, which is calculated by using Empirical Orthogonal Function (EOF) applied to the monthly mean 1000-hPa height anomalies poleward of 20° latitude for the Northern Hemisphere [18].

There is a great interest in researching the ocean responses, such as variations in sea surface temperature (SST), to the atmospheric oscillations, although much debate exists on the mechanisms of how they are interacted. [19]–[20]. Much interest on the correlation between the SST and NAO (and AO per extension) lies in that it gives information on the coupling between atmosphere and ocean [21]–[23]. In general, both climatic indexes could have an influence on SST. The response of SST to the changes of NAO was described in Visbeck [22], while no report on that to the AO was documented yet [14].

Frias et al. [24] analyzed the impact of the NAO and AO on the Iberian water resources, and they observed that NAO could explain the inter-annual variability of southern Iberia, while AO was better associated with river flow in the northern basins. This suggests that NAO and AO may have different effects on the cycling of the freshwater runoff, which might have a delayed effect on SST in the eastern Mediterranean Sea.

In this line of reasoning, the aim of this study was to explore the possible combined, differential and delayed effects of the NAO and AO on the inter-annual SST variation in the Alborán Sea, and to discuss possible mechanisms for these effects based on their impact on freshwater runoff. The principal novelty is that we analysed the effects of NAO and AO separately and together.

## Materials and Methods

### Study area

**Figure 1:**
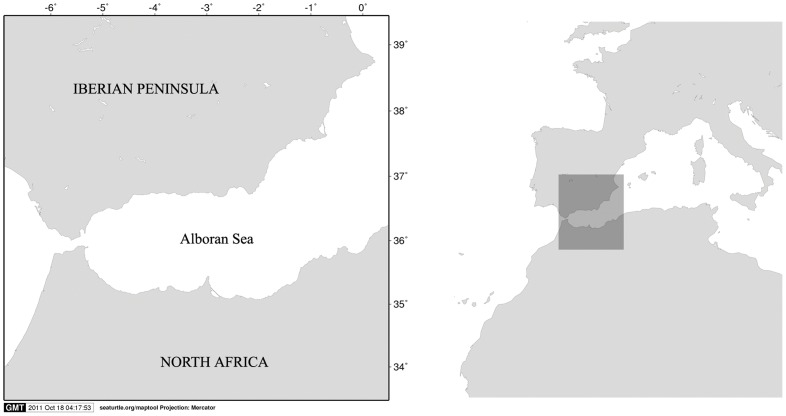
Geographical context of Alborán Sea.

From an oceanographic point of view, the Mediterranean is a peculiar sea, because the important oceanic events are occurring at a small scale [25]. In this context, the Alborán Sea (Figure 1) (the westernmost basin in the Mediterranean Sea) is the frontier with the Atlantic Ocean; here, the surface (less saline) Atlantic waters entering the Mediterranean, and deep (more saline) Mediterranean waters leaving the Mediterranean converge. The Alborán Sea basin, which is considered as a channel bordering to the north with Spain and to the south with Morocco, is a transitional zone between the Atlantic and Mediterranean waters [26]. The Atlantic Current in the Alborán Sea traces two anticyclonic gyres where surface waters accumulate. This general circulation pattern creates a strong frontal system known as the Alborán Sea front. Many authors have reported an Alborán–specific upwelling process [27] which roughly coincides with the northern boundary of the Atlantic current and is evidenced by strong thermal, haline and trophic north–south gradients. Moreover, the oceanography of the Alborán Sea responds to changes in the anticyclone of Azores [26]. Sea surface temperature from Alborán Sea is a complex variable that is also influenced by factors other than downwelling and upwelling water masses. However, the annual average value of SST shows a low deviation in the Alborán Sea.

### Data sources

#### Sea Surface Temperature

Monthly SST averages for the Alborán Sea, for twenty-nine years (since 1982 to 2010, the whole period available) were extracted from the Extended Reconstruction Sea Surface Temperature (ERSST.v3b) dataset, freely available from the National Oceanic and Atmospheric Administration (NOAA website. Available: http://www.noaa.gov. Accessed 2013 March 25).

#### Atmospheric indices (NAO and AO)

Like Frias et al. [24], we used NAO and AO as two independent variables. We used teleconnection patterns in the atmospheric circulation for pressures anomalies based in the normalized pressure to 500 hPa for NAO, and height anomalies at 1000 hPa for AO [6].

Monthly NAO index values were taken from the website of the National Oceanic and Atmospheric Administration (NOAA website. Available: http://www.cpc.noaa.gov/products/precip/CWlink/pna/nao_index.html. Accessed 2013 March 25).

Monthly AO index values were taken from the website of the National Oceanic and Atmospheric Administration (NOAA website. Available: http://www.esrl.noaa.gov/psd/data/correlation/ao.data. Accessed 2013 March 25).

Changes in NAO trend have a delayed effect on aquatic ecosystems, arguably due to ecosystem inertia [28]–[30]. Hence, we related SST values with the mean annual NAO and AO of the previous year.

The NAO and AO present strong inter-annual and intra-annual variability [2, 8–7], with a strong NAO pattern in cold seasons, primarily from November to March. However, given that annual SST is also influenced by seasonal downwelling and upwelling water masses, it is advisable to analyse the complete year.

### Statistical analysis

Given that the mean annual SSTs in the Alborán Sea only varied about one degree Celsius between extreme values during the study period, it offers little possibility for sound statistical analysis to look for patterns or trends. However, a probabilistic analysis may be introduced by taking a year at random and calculating the probability that the average annual temperature of that year is higher or lower than the average SST for all the years.

Binary logistic regression is widely used for es lishing relationships between environmental independent variables and the probability of response of target variables [31]–[36]. We used a binary logistic regression to estimate the probability to obtain a SST value of a particular year higher than the average SST for all the years. Consequently, we assigned the value 1 when the SST of a particular year was higher than the mean SST of the 29 years pooled together, while we assigned the value 0 when the SST was lower than the mean SST value. The explanatory variables were NAO and AO. We built a model for the NAO and for the AO separately to obtain partial models and then performed forward-backward stepwise logistic regression on both predictor variables to obtain a final multivariate logistic model.

To evaluate the models we assessed their parsimony, goodness-of-fit, and discrimination capacity. We assessed the parsimony of the models using the second-order correction of the Akaike information criterion (AICc) [37], because the ratio between the sample size (n) divided per number of parameters in the function (K) was small [38]–[39]. Model goodness-of-fit was assessed by means of the Hosmer & Lemeshow test. We evaluated the discrimination capacity of our model with the area under the receiving operating characteristic curve (AUC) [40].

Hence, we obtained a predictive model and ranked the 29 analysed years according to their probability of having a mean SST higher than the average. In order to turn the predictive model into an explanatory model, it is necessary to disentangle the different roles of the NAO and AO in the final predictive model, because they are correlated [41]–[42]. We performed a variation partitioning procedure to specify how much of the variation in ranking the years according to the final model was explained by the pure effects of NAO and AO, and which proportion was attributable to their shared effect [42]–[43]. The part of the variation in ranking the years in the final model explained by each variable (R^2^_NAO_ and R^2^_AO_) was obtained by correlating the ranks obtained from the final model and the partial models, using Spearman rank correlation coefficient and the squared correlation values. Then, the pure independent effect of each variable (R^2^_pNAO_ and R^2^_pAO_) was assessed by subtracting from 1 (the whole variation) the variation explained by the other variable (R^2^_pNAO_ = 1-R^2^_AO_, R^2^_pAO_ = 1-R^2^_NAO_). The variation attributable to both factors acting collaborately (R^2^_NAO+AO_) may be obtained by subtracting from 1 the pure effect of the two factors (R^2^_NAO+AO_ = 1-(R^2^_pNAO_+R^2^_pAO_)) [43].

To test the seasonal variability in SST, NAO and AO we calculated the average of each of these variables for the winter season (January, February and March), spring (April, May and June), summer (July, August and September) and autumn (October, November and December). In a first step, we decomposed each yearly variable analyzed (SST, NAO and AO) in its main seasonal components. Thus, we calculated, based on forward-backward stepwise binary logistic regression, the probability of getting a SST value of a particular year greater than the average SST for all the years, using as explanatory variables: mean winter SST (SSTwinter), mean spring SST (SSTspring), mean summer SST (SSTsummer), and mean autumn SST (SSTautumn). We did the same analysis for the NAO and the AO. Given that SST fluctuates intra-annually, we also used logistic regression to investigate the seasonal NAO and AO values which may affect SST of subsequent years and seasons.

### Meteorological interpretation

The differential effects of NAO and AO on the Alborán Sea SST could be due to two different aspects in which NAO and AO indices differ: the statistical way of reducing the signal (EOF *vs* RPCA), which should not be relevant for our model to be sound, and the level of used data (surface-1000 hPa for AO and midtroposphere-500 hPa for NAO), where we believe the differences lie. To test this hypothesis we correlated the annual mean of geo-potential at 1000 and 500 hPa with the mean annual NAO and AO indices of the previous year for the period 1948 to 2007.

## Results

### Annual SST

Mean annual SST during the study period was 18.715°C, with a range of 1.063°C (18.097°C –19.16°C), and 0.284°C of standard deviation. When we analysed the effect of NAO and AO separately on SST we obtained a significant partial logistic regression model only for the NAO (χ^2^ =  4.044, df =  1, *p* =  0.0443) with no significant difference (Hosmer & Lemeshow *p* =  0.590) between predicted and observed values, acceptable discrimination capacity (AUC = 0.735) [44] and an AICc value of 39.754. The parameter for NAO was negative, so the higher the annual NAO index a determined year the lower the probability of getting an annual SST value higher than average the following year.

**Figure 2:**
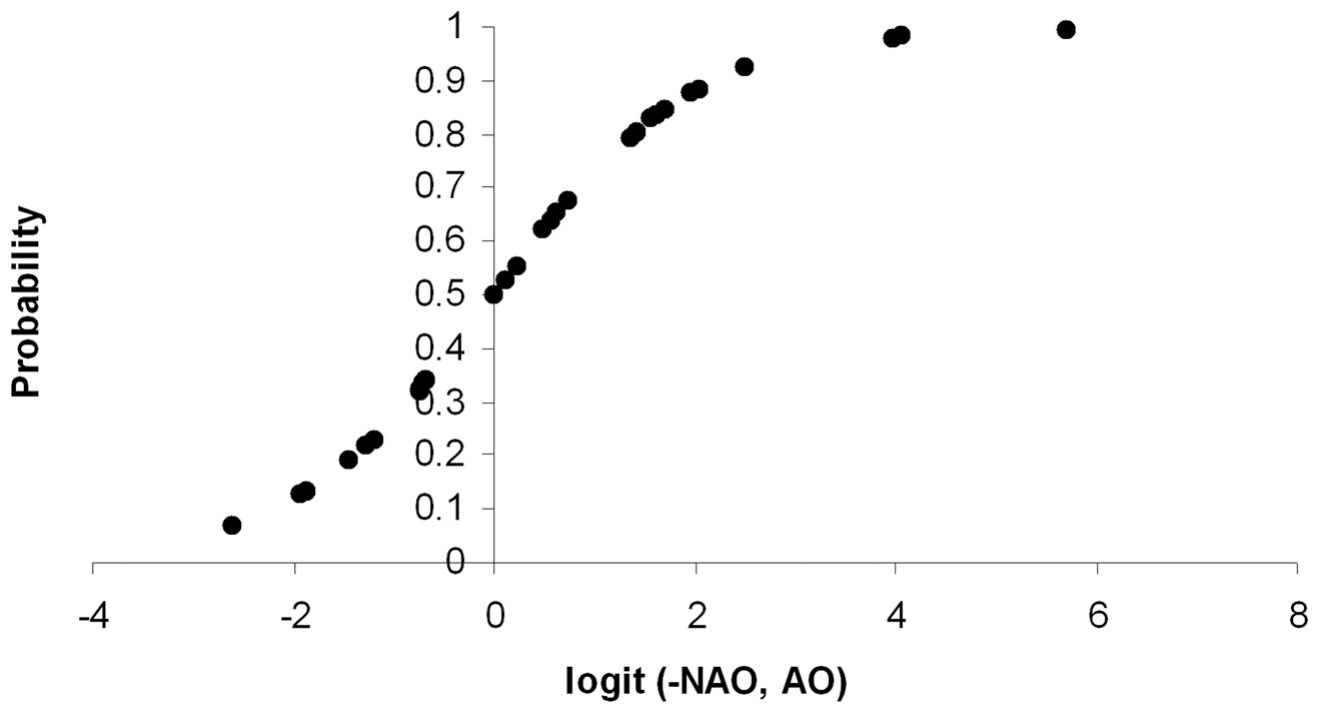
The probability of obtained a SST value higher than the annual average in the study period pooled together versus the logit function (y) from logistic regression using North Atlantic Oscillation (NAO) and Arctic Oscillation (AO) as predictive variables.

When we analysed the effect of NAO and AO together on SST we found that the probability of observing mean annual SST values higher than average was significantly (χ^2^ =  11.694, df =  2, *p* =  0.003) related to NAO and AO values of the previous year. The effect of NAO on SST was negative, while that of AO was positive. The logit function (y) derived from logistic regression presents the form (Figure 2):
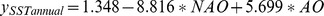



All parameters in the function were significant according to the Wald test (p<0.05). Model goodness-of-fit statistics indicated a good fit of the model to the data, as no significant difference (Hosmer & Lemeshow *p* =  0.585) existed between predicted and observed values, and the AUC of the model was 0.838, which can be considered excellent discrimination [44], [45]. The AICc of the combined model was 34.602, much better than that of the partial model.

**Figure 3:**
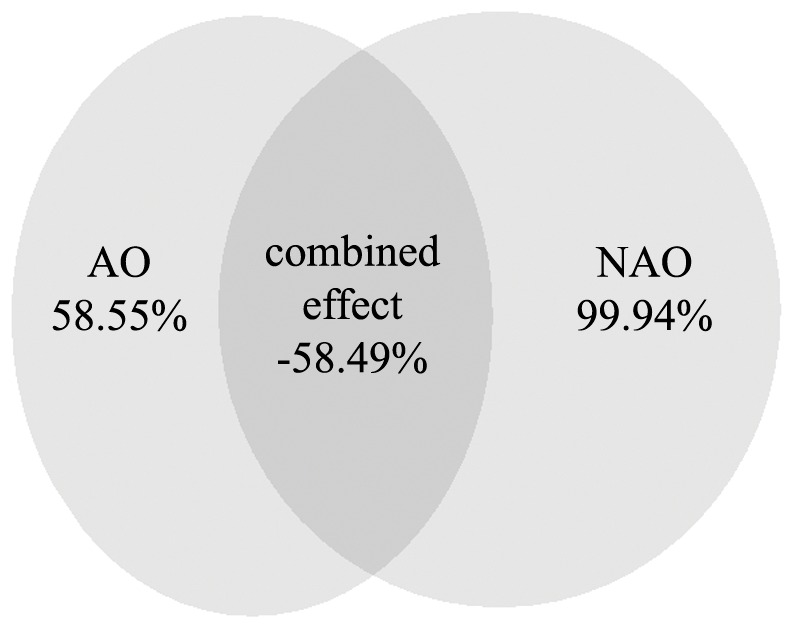
Variation partitioning of the final model based on the NAO and AO indices combined. Values shown in the diagrams are the percentages of variation of the final model explained by the partial models based on the two variables separately.

The values of the NAO and AO indices during the study period were positively correlated (Spearman correlation coefficient NAO-AO =  0.682; *p* =  0.0000457; n =  29). The assessment of the relative explanatory power of NAO and AO in the final model was represented in Figure 3. The pure effect of NAO explains 99.94% of the variation of the ranking of the years in the model, while the pure effect of AO explains 58.55% of that variation. The shared effect of both variables explains−58.49% of the variation of the ranking of the years according to model values, which denotes the degree to which the effect of one variable obscures the effect of the other, given that the sign of the shared effect is negative [46].

**Figure 4:**
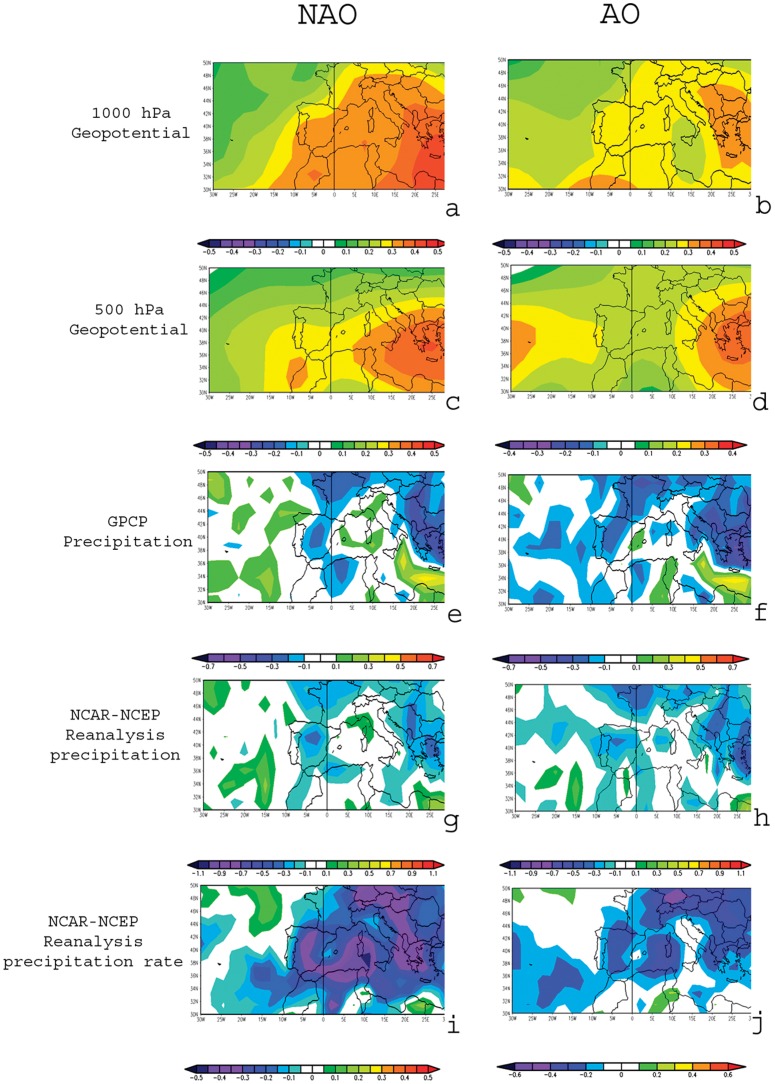
Pearson Correlation coefficients of several annual mean meteorological variables with the mean annual North Atlantic Oscillation (NAO) and Arctic Oscillation (AO) indices of the previous year for the period 1948 to 2007. Key: GPCP, Global Precipitation Climatology Project; NCAR-NCEP, the National Center for Atmospheric Research and the National Centers for Environmental Prediction (NCEP).

We show in Figure 4a-d the annual mean correlation of geopotencial at 1000 and 500 hPa with the annual mean NAO and AO indices of the previous year for the period 1948 to 2007.

### Seasonal SST

When decomposing SST in seasonal values, we observed that the variation in mean annual SST was due mainly to variation in the mean winter SST, and then to variation in the mean summer SST (in this order), with no additionally significant contribution of the mean autumn SST and mean spring SST, according to the logit equation that we show in Table 1.

**Table 1:** Significant logit functions obtained when assessing seasonal effects.

Logit function	χ^2^ (p<0.05)	AUC	AICc
y_SSTannual_ = 338.674*SSTwinter+191.538*SSTsummer – 9609.926	39.336	–	–
y_SSTannual_ = −1.9*NAOautumnpy+0.362	7.119	0.787	32.679
y_SSTwinter = _−1.669*NAOautumnpy+0.182	5.995	0.748	34.359
y_SSTsummer = _−1.45* AOautumnpy−0.079	5.878	0.752	34.752
y_NAOannual_ = 143.043*NAOautumn+148.712*NAOwinter+122.009*NAOspring – 72.143	40.168	–	–
y_AOannual_ = 81.221*AOautumn+71.85*AOwinter+81.195*AOspring+ 79.861*AOsummer+4.428	40.168	–	–

SSTannual: mean annual SST, NAOannual: mean annual NAO, AOannual: mean annual AO, SSTwinter: mean winter SST, SSTsummer: mean summer SST, NAOwinter: mean winter NAO, NAOspring: mean spring NAO, NAOautumn: mean autumn NAO, NAOautumnpy: mean autumn NAO in previous year, AOwinter: mean winter AO, AOspring: mean spring AO, AOsummer: mean summer AO, AOautumn: mean autumn AO, AOautumnpy: mean autumn AO in previous year, χ^2^: Chi-squared for the model, AUC: area under the receiving operating characteristic curve, and AICc: Akaike information criterion corrected.

However, the variation in mean annual NAO was explained by variation in the mean autumn NAO, mean winter NAO, and mean spring NAO (in this order) (see Table 1), while variation in annual AO was explained by the mean autumn AO, mean winter AO, mean spring AO and mean summer AO (in this order) (Table 1).We found that variation in mean annual SST and mean winter SST could be significantly related to the mean autumn NAO of the previous year (Table 1), while mean summer SST was related to mean autumn AO of the previous year (Table 1).

## Discussion

### Comparisons between NAO and AO Effects

#### Mathematical Interpretation

According to AUC value, the best model to explain the annual SST variability was the model that includes the annual NAO and AO. The AO effect on SST appears to be totally obscured by that of the NAO (Figure 3). Mathematically, the correlation between two factors may obscure the contribution of each of them to an effect [46]–[48]. This is particularly true for the correlations among three variables when two of the pair-correlations are positive and the other is negative [49]. In this study, the AO is positively related to SST and NAO, but the NAO is negatively related to SST. In this situation, the real relationships between the variables only arise when the three variables are analysed together. Figure 3 shows the degree to which the effect of the NAO and the AO obscure each other. This reciprocal obscuring of effects is due to the positive correlation between NAO and AO and their contrary effects on SST. For example, high values of AO favour an increase in SST, but this effect tend to be offset by the detrimental effect of correlated high values of NAO on SST. This is why a direct significant relationship between AO and SST could not be found. Only when the effect of the NAO has been already contemplated, the AO manifest a significant relationship with the residuals of SST, i.e., for a given value of NAO the AO has a significant effect on SST residual variation.

NAO and AO tend to be correlated. However, NAO and AO were not correlated (r = 0.413, p = 0.235, n = 10) during the five years with the highest probability and the five years with the lowest probability of having an annual SST higher than average. Moreover, in the five years with highest probability of having a higher than average annual SST, there were two years with negative mean NAO and positive mean AO (both with a mean SST higher than average, Table 2). In contraposition, two of the five years with lowest probability of having an annual SST higher than average presented positive mean NAO and negative mean AO. Consequently, in our final model the NAO and the AO are two related parameters with combined but sometimes opposed effects on the SST in the Alborán Sea.

**Table 2:** Corresponding SST (°C), North Atlantic Oscillation in a previous year (NAOpy), and corresponding Arctic Oscillation in a previous year (AOpy) as well as probability from logistic regression estimated for the combine model.

Year	SST	SSTbinary	NAOpy	AOpy	Probability
1987	18.589	0	0.503	0.085	0.069
1985	18.707	0	0.248	−0.192	0.127
2005	18.712	0	0.243	−0.192	0.132
2000	18.785	1	0.391	0.113	0.189
1993	18.115	0	0.581	0.437	0.217
1984	18.097	0	0.310	0.032	0.231
1983	18.547	0	0.430	0.298	0.322
2001	18.810	1	0.207	−0.046	0.323
1995	18.943	1	0.576	0.532	0.333
1988	18.470	0	−0.123	−0.544	0.338
1986	18.399	0	−0.183	−0.519	0.501
1992	18.228	0	0.268	0.197	0.526
1994	18.594	0	0.179	0.079	0.555
1996	18.731	1	−0.081	−0.275	0.622
1990	19.012	1	0.702	0.950	0.640
1997	19.134	1	−0.214	−0.456	0.654
1982	18.445	0	−0.213	−0.435	0.678
2008	18.755	1	0.173	0.269	0.794
2004	18.960	1	0.098	0.152	0.795
2003	19.022	1	0.039	0.072	0.804
2006	19.162	1	−0.268	−0.375	0.828
2010	18.935	1	−0.243	−0.330	0.834
1989	18.796	1	−0.013	0.040	0.845
1991	18.432	0	0.594	1.024	0.875
2002	18.833	1	−0.183	−0.162	0.885
1998	18.812	1	−0.157	−0.040	0.924
2007	18.981	1	−0.208	0.138	0.981
1999	18.754	1	−0.481	−0.271	0.983
2009	18.986	1	−0.378	0.177	0.997

We order the data according to their probability (low to high) to have a year with a SST annual greater than average SST of the study period (SST mean study period =  18.715). Furthermore, in the SSTbinary column we show the year that they had a SST annual greater than the average SST of the study period.

Our seasonal analyses showed that our annual values reflect mainly the variation at the beginning of the year for SST and at the end of the year for NAO and AO. In this way, the lag in the effect of climatic conditions on SST is in fact shorter than a year. In fact, in terms of the difference in AICc our model based on the NAO in autumn of the previous year was better than that based on the NAO and AO of the whole previous year, although the calibration, the amount of variability in SST explained, and the discrimination capacity were all higher in the model including NAO and AO. In addition, the effect of the seasonal AO on the seasonal SST seems to be more delayed than that of the NAO.

#### Meteorological Interpretation

There are positive correlations for both AO and NAO with 1000 (Figures 4a and 4b) and 500 hPa fields (Figures 4c and 4d) for all the Mediterranean area, but there is an important difference in the correlation patterns for 500 hPa in the Gulf of Cadiz, close to the North African Atlantic coast. There are significant positive correlations (higher than 0.4) over the Gulf of Cadiz between NAO index and 500 hPa geopotential (Figure 4c) which are not found in the correlation patterns with AO (Figure 4d). This signal can be interpreted as meaning that when the mean annual NAO index is negative, for the following year there is a likelihood of negative anomalies in 500 hPa geopotential without a significant corresponding result in 1000 hPa. This signal is not equivalent for the AO index, a result which can be directly attributed to the different methods of calculating AO and NAO indices. In other words, the AO index used in this study, because of being calculated with surface data, is unable to detect signals which occur only in the mid-troposphere.

The question now lies in identifying a meteorological system producing precipitation in our area of interest (SE Iberian Peninsula and Alborán Sea) with negative anomalies of geopotential in mid-troposphere but without significant geopotential anomalies at surface levels. The single structure that answers this question is the so-known cut-off low system. A cut-off low pressure system represents a closed low in the upper and mid troposphere that has become completely detached (or “cut off”) from the characteristic westerly current of the jet stream [50]–[52], and which is usually advected towards the equatorial side of the mid-latitude westerlies. Its intensity is higher in the upper troposphere, decreasing downwards and it even being possible to find anticyclonic circulation at the surface. Systems related to cut-off low pressure are capable of affecting the weather conditions at the earth's surface to a considerable degree for periods of several days at a time. The instability of the troposphere beneath the cut-off low pressure system can lead to the occurrence of severe convective events, depending on surface conditions. Cut-off low pressure systems yield significant precipitation when the air mass below the cut-off low pressure system is very moist and generates a potentially unstable condition. Such weather systems are among the most severe that affect the Mediterranean and are responsible for some of the most catastrophic events in terms of their precipitation rate, especially during the warm seasons [53]. In fact, studies in the region where the occurrence of cut-off low pressure system are common [50], show that the highest intensity of cyclonic-related precipitation is located at a distance of 300–400 km from the centre of the cut-off low pressure system [54]. Convective rainfall occurs within about 300 km of the cut-off low pressure system centre, with a pronounced peak close to the centre of the cut-off low pressure system that is displaced marginally eastwards (i.e. in front) of it. This region of convective precipitation coincides with an area of reduced stability. At the same time, large-scale precipitation is distributed along the east–west axis that passes through the cut-off low pressure system. In about 50% of cases, it is this precipitation that dominates. A scheme of the cloudiness and precipitation associated to a typical cut-off low pressure system is displayed in Nieto et al. [55].

The high positive correlation of NAO index with 500 hPa geopotential over the Gulf of Cadiz in Figure 4c is clearly related with the likelihood of occurrence of cut-off low pressure systems over this area. Placing the precipitation patterns associated to the cut-off low pressure system conceptual model over this area of high correlation, it is possible to view the likelihood of intense precipitation in the SE of the Iberian Peninsula and the Alborán Sea linked to cut-off low pressure systems over the Gulf of Cadiz. The study of Nieto et al. [55] on precipitation over the Iberian Peninsula linked to cut-off low pressure systems [55] showed the important precipitation in the area associated with cut-off low pressure systems that occur over the Gulf of Cadiz. Returning to differences between NAO and AO indices, these results should be reflected in the correlations of these indices with precipitation in the SE Iberian Peninsula and the Alborán Sea. Figures 4e to figure 4j clearly show the higher correlation of NAO index when compared with the AO index for both precipitation (Figures 4e-h) and precipitation rate (Figures 4i-j) in this area. These factors support our hypothesis and justify the higher influence of NAO on SST in the Alborán Sea.

### Environmental integration

Recent papers discussed large-scale climate variability for several marine ecosystems and suggested types of ecosystem responses to climate [15]. Visbeck et al. [22] reported negative and significant correlations between winter-SST and NAO in the Mediterranean Sea and the Gulf of Cádiz. When the NAO is negative, runoff from the Iberian Peninsula and around the north-western Mediterranean basin increases [28], [56]–[59]. It is then possible that the increase in SST in the Alborán Sea in response to the negative NAO could be mediated by the increase in the input of continental freshwater, although other indirect mechanisms are possible, given that atmospheric general circulation models and trace-gas compositions display NAO-like fluctuations [22], with widespread effect in the North Atlantic area. In any case, this kind of effect should be noticeable every year, and our results suggest that this is the case, only that the opposed effect of the AO partially obscures that of the NAO.

The Alborán basin presents the peculiar shape of a funnel, surrounded by a rugged coastline, with the highest peaks of the Iberian Peninsula (eg Mulhacen and Veleta peaks over 3000 meters high), and where the mountains accumulate snow. Thus, the accumulated snow is an important fresh-water reservoir. The one year delay in the effect of the NAO and AO on the SST could be partially related with the amount of accumulated snow. To test this hypothesis we correlated the total snow in the North Alborán watershed for a year with the annual average SST of the subsequent year. To do this we used the values of snow gauge [60] from 1996 to 2009 (Table 3). We obtained a significant Pearson correlation (r =  0.535; p =  0.04).

**Table 3:** Accumulated snow (l/m^2^), and mean SST (°C) in of the subsequent year.

Previous-years	Accumulated snow	Mean SST in subsequent year
1995	312.2	18.731 (year 1996)
1996	1385.2	19.134 (year 1997)
1997	742.2	18.812 (year 1998)
1998	478	18.754 (year 1999)
1999	706.4	18.785 (year 2000)
2000	978.7	18.8096 (year 2001)
2001	736.9	18.833 (year 2002)
2002	977.4	19.0218 (year 2003)
2003	1379.9	18.959 (year 2004)
2004	748	18.712 (year 2005)
2005	802.6	19.162 (year 2006)
2006	1069.2	18.981 (year 2007)
2007	865.3	18.755 (year 2008)
2008	1725.2	18.986 (year 2009)
2009	1998.8	18.935 (year 2010)

The snow thaw could modify the SST in the Alborán Sea due to its effect on the mixed layer, as an increase in freshwater runoff from snowmelt could maintain the mixed layer at a higher depth. Thus, we related the depth of the mixed layer with the amount of snow in the southern basin of the Iberian Peninsula. We calculated the mixed layer depth based on the changes of temperature and density with reference values [61]. We used the temperature criterion proposed in [61], by considering the mixed layer as the depth where the temperature change is 0.5°C at least, with respect to the surface temperature. Sea Temperature data were obtained from the Simple Ocean Data Assimilation (SODA). Reanalysis data covering the period 1958–2008 are available at monthly scale with a horizontal resolution of 0.5°×0.5° and a vertical resolution of 40 levels (SODA website. Available: http://www.atmos.umd.edu/~ocean/. Accessed 2013 March 25). For detailed information about the methodology the reader is referred to [62], [63]. An overview of different methods to calculate the mixed layer can be seen in [64]. The mean mixed layer was calculated for spring (April, May and June) and summer (July, August, September) over the period 1996-2006, and was used to compare with snow data corresponding to the previous winter. Five points located along the southern coast of the Iberian Peninsula (at latitude 36.25°N and longitude ranging from 355.25 to 357.25 °E) were considered in the present study. At mid-latitudes in the Northern hemisphere there is a marked seasonal cycle of the mixed layer evolution, consisting of a deepening of the mixed layer during the winter and the development of seasonal thermocline during the summer [65]. In the case of the Alborán Sea, the mixed layer is quite shallow with ranges of ∼10 m in Spring-Summer to ∼20 m in winter [66]. Although there are several sources of energy that can affect the formation of the mixed layer, wind-driven currents and convection induced by increase of surface density is considered to be the main mechanisms in the study area. Normally, during summer, solar heating of the near surface leads to more stable density stratification which, in turns, inhibits the penetration of wind-induced mixing. According to the data estimated for the north of Alborán Sea (Table 4), we found a markedly negative correlation between the depth of the mixed layer in spring and the snow accumulated during October to December of the previous year (r =  -0.847, p =  0.001). The negative correlation between accumulated snow and mixed layer (the mixed layer becomes more negative when the amount of snow during the previous year was over the mean) seems to indicate that the snow melting during Spring-Summer drives an extra amount of water on top of the coastal water, which modifies the stability of the water column and results in the deepening of the mixed layer.

**Table 4:** Corresponding SST (°C), Mixed layer depth, and accumulated snow (l/m^2^) for autumn in previous year (October, November and December), and accumulated snow (l/m^2^) for winter (January, February, March) estimated for the north Alborán Sea for the period 1996–2006.

Year	SST	MLD	Snowautumn	Snowwinter
1996	18.731	−12.343	184.3	1117.8
1997	19.134	−14.510	340	365.1
1998	18.812	−12.677	255.6	240.9
1999	18.754	−10.454	235.8	452.6
2000	18.785	−18.343	419	167.5
2001	18.810	−10.788	289	395.5
2002	18.833	−14.288	275.3	421
2003	19.022	−22.010	513.1	728.8
2004	18.960	−25.066	383.9	119.5
2005	18.712	−8.232	126.7	628.9
2006	19.162	−7.732	211.2	727

Consequently, an explanation for the role of the AO in the final model could be that a positive AO, which implies a colder atmosphere in the Polar Regions, could favour occasional cold waves over the Iberian Peninsula which, when coupled with precipitations favoured by a negative NAO, may result in snow precipitation. This snow may be accumulated in the high peaks and melt down in spring-summer of the following year, which consequently increases the runoff of freshwater to the sea, causing diminution of sea surface salinity and density, and blocking the local upwelling of colder water. This could also help in explaining the opposite effect of NAO and AO on the SST of the following year. In fact, 75% of the years (9 years out of 29) with SST lower than average were preceded by years with positive NAO that caused low precipitation and low snow accumulation. In addition, 3 of the years with SST higher than average (1989, 2007 and 2009) were preceded by years with negative NAO and positive AO that in the latter two years resulted in a high quantity of snow accumulated in peaks. Note that we did not consider the effect of snow for the year of 1988, due to lack of data.

A remarkable feature of the NAO is that its centre of action, the Icelandic Low and the Azores High, has shifted considerably to the northeast [67]. The Low NAO is now over the Barents Sea, and the High NAO is near the southwest corner of Ireland. Therefore, the actual trend could affect the future regional impact of the NAO, and in consequence its effect on the SST in the Alborán Sea.

Many embryonic and larval phases of animals are correlated with high sea temperatures [68]–[72]. Our results suggest that any change in NAO and AO could affect the nutrient fluxes and modify the SST, which could in turn affect the marine biodiversity in the Alborán Sea. Further research is needed in the correlated patterns of the atmosphere and the ocean, and the derived consequences for marine biodiversity as well.
